# The Air as an Anæsthetic

**Published:** 1876-05

**Authors:** W. G. A. Bonwill

**Affiliations:** Philadelphia.


### ARTICLE V.

### *The Air an Anæsthetic.*

BY W. G. A. BONWILL, D. D. S., PHILADELPHIA.

Read before the Franklin Institute, Wednesday, November 17th 1875.

In presenting for the first time this very important discovery of the remarkable effect of ordinary air upon the human system as an obtunder of pain, I shall not attempt to go into the cause or *modus operandi* of this simple gas, but shall in a future article take up this point and endeavor to show why it is capable of such effect; and its uses as a harmless agent in producing far greater good than hitherto "dreamed of in *air* philosophy."

To what extent it may be carried in producing complete anæsthesia I cannot now say, nor would I like to assert or prognosticate its future. Enough for me to-night to inform you of my limited experience with it. In so doing I but feel that some will in ridicule turn aside without having for *one minute* made the effort to prove what *I have found true.*

So far as my reading and research have gone I have never seen the least hint at such properties as I claim. It may be recorded somewhere, but I have never seen it. Nor have I found the first one, of those to whom I have spoken regarding it, who has been able to recall anything that would tend to throw the least light upon it. So, whether I am the first to place it on record remains to be seen. I do so with no other motive than the good of my fellows, caring not for any ridicule that may be heaped upon me, since the eight years that I have just passed in the world of invention has taught me to regard but one thing: "If thou hast a truth to utter, speak! and leave the rest to God." As a foundation, truth for future investigation is all that I can at present do.

As *Air* and its power as a motor has been so fully discussed of late in connection with "The Keeley Motor," I beg of you to not confound me with a similar enterprise—not that I would ridicule Mr. K for a moment, but would rather lend him or *any one* engaged in developing new truths, a helping hand. In my case I give you all the secrets of its peculiar powers for assisting in obtunding pain, that anyone may satisfy himself to his heart's content. For twenty-one years in dental practice I have been looking for simple means of alleviating the suffering of my patients, and in giving to my brethren in practice such assistance in machinery as would save to them time, labor and pain.

My first effort was electricity, which did not fully satisfy my designs. Ether and chloroform would produce this desired effect in extracting, but these latter agents could not be used for so prolonged a time as was requisite in the removing of decayed tooth structure preparatory to filling. Electricity would in many cases obtund, but it was not always easily practiced. It was *not what I wanted*, and my mind was open to the next best thing that would offer.

By what means I know not (so long ago has it been) that led me to use air as I now propose, other than the observing of the action of a human subject in pain, which was of hourly occurrence in my office. All of you have noticed that when a finger has been badly hurt, the involuntry act is to place it at once to the mouth, and forcibly draw the air into the lungs and hold it a moment, and again repeat it, which truly gives relief in many minor cases. On the other hand, you may have noticed a person hurt, instead of inhaling a large body of air rapidly into the lungs, a cry or loud yell is set up. Such suffer far more severely. Tears do very well in grief, but not for extreme bodily pain.

Well, having observed the good effects of the involuntary efforts of one that is in pain from the inflating of the lungs,

I was led to adopt it in my dental practice, and for at least sixteen years I have used it in such manner as I shall show and with such success, that I have been led by my association with it to carry it a step further. My usual plan has been to have my patients forcibly inhale the air just at the very instant I was making my thrust with the excavator to remove very sensitive tooth bone, and enjoin them to keep up the action as they felt my movements, which they soon understood, as they were made in such cases with greater regularity, that the patient might know what to expect.

That such a plan was worthy of adoption needed no further argument than to see the differnnce in the patients and their expressions as to its effects. Within the past six months my mind has been specially dwelling u, on this subject; and, while taking a walk with my children one Sunday afternoon, it was *vividly* presented to me that *air must have anæsthetic properties when taken into the lungs in larger quantities than the blood requires for oxygenation.* I reasoned thus: If the forcible and occasional inhalation could produce the phenomena which I had noticed so long in obtunding sensitive bone, and as nitrous oxide, which is richer in oxygen than air, produces such wonderful effects, why not the pure air we breathe, if rapidly and continuously taken into the lungs for, say, half a minute or more, produce a still more marked effect upon the brain than as I had before used it? The heart pulsating at sixty to the minute, requires about fourteen inhalations to the minute to supply the blood with the exact amount of oxygen, with an occasional "long breath" to meet any special demand.

Now, that the automatic movements are fixed by law that there shall always be a reciprocity of action between the number of pulsations and inhalations, whether sitting still or in making violent muscular movements, what effect will be produced upon the brain and nerve centres if, while the heart is beating sixty to the minute, I inhale three, four or five times the quantity of air into the lungs, supplying the blood with far more oxygen than it requires, and nitrogen in proportion. Such, while walking along, was the course of reasoning, and I at once commenced a rapid inhalation, inflating my lungs to their fullest capacity each time, when, in twenty seconds, I found such marked effect upon my locomotion that I was obliged to stop the inhalation. My reasoning was correct, that when air is inhaled, as I had before agreed to myself, an effect was produced similar to anæsthetics; I hurried home and had a lady, living with me, to stand up and make inhalation as I would show her; and, to my gratification, she soon had to take her chair from the very early effect.

Scarcely a day since but I have tried the experiment upon many of my patients, and know of no case of failure, but the patient is enabled to bear in conscious silence (other than the rapid breathing,) the otherwise very painful operation of cutting sensitive tooth-bone.

In the extracting of teeth I have not had much experience, as it is ssldom I sacrifice even an old root; but have no doubt its effects might be such, from prolonged inhalation, as to, in simple cases, answer all the purposes as an anæsthetic. Not satisfied in the statements of my patients, I have again and again repeated it upon myself with such strong and decided effects, that although evident to my own senses of some marked change in my condition, I could hardly realize, by reason and practice, that *such an effect* could come from the *pure air* which we have always been taught is capable only of giving life and power. Last evening I had several persons stand up and go through the rapid movements, and every one was affected to a very marked degree, especially my wife, who positively assured me that she was incapable of offering resistance, although conscious of what was passing. There was that pricking sensation in the lips, such as is common from inhaling other anæsthetics. She repeated it, as did the others, and any one could see the difference in the countenance, and they all assured me it was no mistake, but the effect was sure if the air was taken as prescribed. I cannot be deluded in this matter. I have tried to believe it was not so, but I am instantly convinced by the rapid inhalation, from twenty to thirty seconds.

A beautiful and forcible illustration of the effect of air occurred a short time ago in the case of an elephant suffering from a disease of the eyes. The physician advised the application of nitrate of silver. The animal not knowing beforehand how much pain it would occasion, was unprepared to meet the very sudden violent pain which ensued, and manifested his anger toward the doctor. The application proved so successful that when he was brought out to receive a second application, his demeanor was very much changed towards the doctor, and he laid lown at once, and just as the application was about to be made, the animal took one long inhalation and held it until all was over, when he allowed the breath to pass gradually from the lungs without any signs of pain.

Another case bearing upon it: Who has not noticed the giddiness produced after blowing a fire for a few minutes? I had attributed it to stopping over, but now I see more clearly the philosophy of it—the larger amount of air passing into the lungs than is necessary for oxygenation.

You may ask of what use it is to mankind? To the dental world alone it makes the weary hours, spent in great pain, as dentistry is ordinarily practiced, pass with far more pleasure. As a remedial agent, when thus taken mechanically, who can place its bounds. Any one, in any place, at any time, under any and all circumstances, can use this heaven's gift, in satisfying himself or herself, that it certainly has an effect hitherto unnoticed, call the phenomena by what name you will.

I *record the fact*, and shall hope to minutely investigate it in the future. While penning these lines I have stopped to try its effects again upon myself, and I must reiterate everything I have heretofore said in regard to it, and still ready to doubt to the last, notwithstanding my senses indicate the change undergone. Whether more marked effects could come of rarified air by heat, or compressed air, hot or cold, breathed through a mouth-piece, similar to nitrous-oxide apparatus, can only be told by further research. Whatever may be done in the future in investigating this peculiar quality of air, let it be done not so much to disprove what I may have divulged, but let it be a centre of thought for radiation, that greater truths may come of what has here been unselfishly presented.—*Penna. Journal of Dental Science.*

